# Extent of podoconiosis-related stigma in Wolaita Zone, Southern Ethiopia: a cross-sectional study

**DOI:** 10.1186/2193-1801-3-647

**Published:** 2014-11-01

**Authors:** Abebayehu Tora, Hannah Franklin, Kebede Deribe, Ayalu A Reda, Gail Davey

**Affiliations:** Department of Sociology, Wolaita Sodo University, Sodo, Ethiopia; Brighton and Sussex Medical School, Brighton, UK; Population Studies and Training Center, Brown University, 68 Waterman St, Providence, RI USA

**Keywords:** Podoconiosis, Enacted stigma, Felt stigma, Ethiopia

## Abstract

Studies have indicated that social stigma related to podoconiosis (endemic non-filarial elephantiasis) has a major impact on the psychosocial wellbeing of patients. However, little effort has been made so far to quantify the level of both felt and enacted stigma in a range of domains of life. We used a recently developed podoconiosis stigma assessment scale to measure levels of stigma as recalled over the previous 12 months. One hundred and fifty patients with podoconiosis rated the levels of stigma they perceived and experienced in ‘interpersonal interactions’, ‘major life areas’ and ‘community, social and civic life’. High levels of stigma were observed on both felt and enacted stigma scales. The overall average stigma score was 40.7 (range 0 to 96). Enacted stigma was scored higher than felt stigma (mean score 21.2 vs. 19.5, respectively). The mean enacted stigma score was higher in ‘major life areas’, and ‘community, social and civic life’ than ‘interpersonal interactions’, while felt stigma was experienced most at the interpersonal level. Over half of patients reported that they had considered suicide in response to discrimination and prejudice, particularly in interpersonal interactions. Forced divorce, dissolution of marriage plan, insults and exclusion at social events were some of the most commonly mentioned forms of enacted stigma reported by affected individuals. Scores for overall level of stigma and enacted stigma increased significantly with stage of podoconiosis while the association observed in relation to felt stigma was only marginally significant (p = 0.085). Appropriate stigma reduction strategies must be identified and implemented in communities highly endemic for podoconiosis.

## Introduction

Podoconiosis (endemic non-filarial elephantiasis) is a geo-chemical disease caused by long-term exposure of bare feet to irritant volcanic soils. Affected people develop disfiguring swelling of the feet and lower legs, and later stages of the disease are characterised by hyperkeratotic skin changes, nodules and lymph ooze (Price 1988). Podoconiosis is one of the Neglected Tropical Diseases (NTDs); chronic disabling diseases that occur in settings of poverty and remain a huge burden in developing countries. The disease is endemic across more than 10 tropical countries in Africa, Central America and Northwest India, and Ethiopia appears to be home to the greatest number of affected people (1 million affected and 11 million at risk of disease). In Wolaita Zone, in southern Ethiopia, over 5% of the population is affected by the disease (Desta et al. 2003). Almost two-thirds of affected people in this area are in the most productive age groups, so the economic burden of podoconiosis is high, with the total costs attributable to the disease estimated at US$16 million annually (Tekola et al. 2006).

Studies conducted in endemic areas in Ethiopia have recorded high levels of misconceptions contributing to stigmatizing attitudes towards podoconiosis affected individuals (GebreHanna 2005; Yakob et al. 2008; Tekola et al. 2009a; Molla et al. 2012). Following Goffman (1963), many authors define stigma as an undesirable or discrediting attribute, reducing an individual’s status in the eyes of society (Link and Phelan 2001; Weiss 2008). The highly insulting term ‘Gedya kita’ [a local term in Wolaita for swelling] is attached to podoconiosis and conveys a devalued social identity for affected individuals (Tekola et al. 2009a, [b]). According to Goffman (1963), some stigmatized attributes are so powerful in the reactions they engender that they are “master status” attributes that become the core identifying attribute of the person who possess them. Podoconiosis, as a disease causing a progressive bilateral swelling of lower legs, gives a “master status” to affected individuals so that they are easily marked and publicly identified in the community (Tora et al. 2011).

The stigma associated with podoconiosis relates to a number of factors: the progressive physical disability that prevents affected individuals from making a living (Desta et al. 2003; Tekola et al. 2006); the misconceptions among community members about the causes of the disease and treatment options (GebreHanna 2005; Yakob et al. 2008; Tora et al. 2011; Ayode et al. 2012; Molla et al. 2012); the burden the disease brings on family members through treatment costs; perceived fear of public identification of familial disease (Tora et al. 2011); and the physical disfigurement caused as the disease advances (Tekola et al. 2008).

Since the consequences of podoconiosis related stigma are clearly visible, it affects patients’ wellbeing and forces various coping strategies with maladaptive outcomes (Tora et al. 2011). The stigma attached to podoconiosis also affects both health-seeking behaviour and achieving effective treatment: in order to avoid negative reactions from others, some podoconiosis patients prefer to conceal their condition, and as a result, their symptoms may worsen (Tora et al. 2011, 2012). The shame surrounding the disease also deters those with podoconiosis from seeking the care of potentially prejudiced health workers (Yakob et al. 2010). Studies revealed that podoconiosis patients are disqualified from full social acceptance, marginalized from participation in social affairs, discriminated against in mate selection and marriage, and have little chance of decision making and leadership roles in the community (GebreHanna 2005; Tora et al. 2011).

Stigma related to podoconiosis can be compared with that related to communicable and non-communicable diseases with obvious physical manifestations. Podoconiosis-related stigma also intersects with that related to diseases with concealed attributes such as HIV/AIDS through the deeply rooted misconceptions related to its cause, transmission and prevention mechanisms. However, in contrast to a number of diseases with stigmatizing attributes such as leprosy, lymphatic filariasis, obesity, mental illness, tuberculosis and HIV/AIDS (Van Brakel 2006; Heijnders and Van Der Meij 2006), very little research has been performed into the extent of stigma related to podoconiosis.

According to USAID (2006), there are many potential benefits of quantifying stigma, including the ability to evaluate anti-stigma programmes, to determine the impact of new services and to detect unintended consequences of a given intervention. However, no attempts have yet been made to formally quantify podoconiosis-related stigma in an endemic rural community in southern Ethiopia. In the present study, we report the extent of podoconiosis-related stigma experienced and perceived by affected individuals across various domains of life.

## Results

### Respondents’ characteristics

Basic socio-demographic information on patients and disease stage is shown in Table 1. Female participants constitute 54%. The majority of participants were above the age of 35, illiterate, and had podoconiosis at a less advanced disease stage.

**Table 1:** **Respondents’ characteristics**

Variables	Respondents (N = 150)	
	Category	Number (%)
Sex	Female	81 (54.0)
	Male	69 (46.0)
Age	<25	23 (15.3)
	25-34	35 (47.0)
	35-49	47 (31.3)
	> = 50	45 (30.0)
Education	Cannot read and write	98 (65.3)
	Can read and write	52 (34.7)
Disease stage	STAGE 2 and below	107 (71.3)
	STAGE 3 and above	43 (28.7)

### Mean scores of felt and enacted stigma as rated by respondents

Complete responses were available for all items of felt and enacted stigma from 120 (80%) patients. As shown in Table 2, the mean (range) scores for overall, enacted and felt stigma were 40.7 (0 to 96), 21.2 (0 to 51), and 19.5 (0 to 45) respectively, where a higher score indicates greater felt or enacted stigma. ‘Major life areas’ such as marriage and employment and ‘community, social and civic life’ were the domains in which enacted stigma was greatest, while felt stigma was most common at the interpersonal level.

**Table 2:** **Mean scores and level of stigmatization**

Stigma scores	Category		
Mean stigma scores (minimum, maximum)	Overall stigma	Enacted stigma	Felt stigma
	40.7 (0, 96)	21.2 (0, 51)	19.5 (0, 45)
Mean stigma scores across domains (minimum, maximum)	*Interpersonal interactions*	5.54 (0, 12)	8.9 (0, 21)
	*Major life areas*	7.0 (0, 18)	6.98 (0, 15)
	*Community, social and civic life*	7.1 (0, 21)	3.4 (0, 9)

### Frequency distribution of levels of stigmatization per items in the scales

Table 3 shows the item-based frequency and percentage distribution of enacted stigma levels per individual items in the scales. Being visited less by friends and avoidance of sharing family members’ household utensils were mentioned by 65.3% and 39.3% of patients. Over 54% of respondents indicated that they had received insults about their feet, while 60% of them reported mistreatment at the workplace. Forced divorce and isolation at social events were also common experiences.

**Table 3:** **Frequency distribution of patients’ responses to 17-item enacted stigma scale**

Domain	Enacted stigma in the last 12 months			
	Yes	Uncertain	No	Total
	N (%)	N (%)	N (%)	
***Interpersonal interactions***				
Deterred from taking part in group activities by others	56 (37.3)	5 (3.3)	87 (58)	148
Family members avoided sharing household utensils	44 (39.3)	14 (9.3)	91 (60.7)	149
Friends spent less time with/visit less because of condition	98 (65.3)	16 (10.7)	35 (23.3)	149
Received insults about foot	82 (54.7)	0 (-)	67 (44.7)	149
***Major life areas***				
Denied a job opportunity because of condition	52 (34.7)	30 (20)	68 (45.3)	150
Forced to leave job because of condition	29 (19.3)	37 (24.7)	83 (55.3)	149
Mistreated at work place	80 (60)	11 (7.3)	49 (32.7)	150
Forced to dissolve marriage plans because of condition	45 (30)	26 (17.3)	75 (50)	146
Divorced because of condition	54 (36)	21 (14)	70 (46.7)	145
Difficult for family members to marry because of condition	63 (42)	12 (8)	74 (49.3)	149
***Community, social and civic life***				
Denied chance of leadership role because of condition	38 (25.3)	19 (12.7)	92 (61.3)	149
Denied chance to make decisions because of condition	36 (24)	22 (14.7)	91 (60.7)	149
Ignored/talked over/told to be quiet because of condition	48 (32)	22 (14.7)	79 (52.7)	149
Unwelcome at community meeting places	74 (49.3)	11 (7.3)	65 (43.3)	150
Isolated at social events	89 (59.4)	9 (6)	51 (34)	149
Not invited to appear at public places	69 (46)	4 (2.7)	76 (50.7)	149
Stared/pointed at while attending social events	71 (47.4)	16 (10.7)	61 (40.7)	148

In the felt stigma scale, as shown in Table 4, over 46% of respondents indicated that they had avoided taking part in group activities with unaffected people. Fifty four percent of patients indicated that they had attempted suicide. The same proportion of respondents also indicated that they had tried to move house as a result of their condition. Over half reported that they had avoided visiting public places and feared that others would feel uncomfortable working with them. Marriage to an unaffected person was feared by 48% and avoided by 46.6% of patients.

**Table 4:** **Frequency distribution of patients’ responses to the 15-item felt stigma scale**

Domain	Felt stigma in the last 12 months			
	Yes	Uncertain	No	Total
	N (%)	N (%)	N (%)	
***Interpersonal interactions***				
Avoid taking part in group activities with unaffected people	70 (46.7)	2 (1.3)	77 (51.3)	149
Use household utensils separately from others	45 (30)	2 (1.3)	99 (66)	146
Feel family are ashamed of them	67 (44.6)	13 (8.7)	69 (46)	149
Avoid spending time with unaffected friends	53 (35.4)	16 (10.7)	77 (51.3)	146
Avoid asking neighbours for help	64 (42.7)	2 (1.3)	82 (54.7)	148
Tried to kill self	81 (54)	3 (2)	66 (44)	150
Tried to change place of residence	81 (54)	1 (0.7)	68 (45.3)	150
***Major life areas***				
Avoid job offer fearing stigma	64 (42.7)	9 (6)	74 (49.3)	147
Fear others will feel uncomfortable working with them	77 (52.3)	5 (3.3)	67 (44.7)	149
Avoid marriage to an unaffected person fearing stigma after marriage	60 (46.6)	13 (8.7)	67 (44.7)	150
Fear marriage to an unaffected person will end in divorce	72 (48)	11 (7.3)	66 (44)	149
Feel condition has affected other family members’ chances of marrying	75 (50)	7 (4.7)	68 (45.3)	150
***Community, social and civic life***				
Feel condition deprives them of playing a leadership role	57 (38)	10 (6.7)	81 (54)	148
Avoid visiting public places	82 (54.3)	4 (2.7)	64 (42.7)	150
Feel able to move around the community freely	37 (24.6)	13 (8.7)	100 66.7)	150

As shown in Table 5, we did not find a statistically significant difference in stigma scores by age, gender or level of education at p-value ≤0.05. However, there was a statistically significant association between level of stigma and stage of podoconiosis, where patients at stage three and above reported higher levels of perceived and enacted stigma. Statistically significant associations were found between stage of disease and total stigma score (p =0.02), enacted stigma (p =0.004), enacted stigma sub-score in community and civic life (p =0.002), enacted stigma sub-score in major life areas (p =0.012), enacted stigma sub-score in interpersonal interactions (p = 0.028) and felt stigma sub-score in community and civic life (p =0.011). The association between stage of disease and perceived stigma score was not statistically significant (p =0.085).

**Table 5:** **Associations between stage of disease and stigma scores**

Type of stigma	Disease stage*	Mean scores (SD)*	F-value	p-value
Enacted stigma	Stage 2 and below	18.9 (14.3)	3.176	0.004
	Stage 3 and above	26.7 (16.2)		
Felt stigma	Stage 2 and below	18.1 (15.6)	0.851	0.085
	Stage 3 and above	23.0 (16.9)		
Total stigma	Stage 2 and below	37.0 (29.3)	2.314	0.021
	Stage 3 and above	49.8 (32.4)		
Felt stigma in major life areas	Stage 2 and below	6.5 (5.9)	0.068	0.140
	Stage 3 and above	8.1 (6.1)		
Felt stigma in interpersonal interactions	Stage 2 and below	8.4 (7.6)	0.192	0.223
	Stage 3 and above	10.1 (7.7)		
Felt stigma in community and civic life	Stage 2 and below	3.0 (2.9)	13.711	0.011
	Stage 3 and above	4.5 (3.7)		
Enacted stigma in inter personal interaction	Stage 2 and below	5.2 (2.7)	0.267	0.028
	Stage 3 and above	6.3 (2.7)		
Enacted stigma in major life areas	Stage 2 and below	6.2 (5.9)	0.892	0.012
	Stage 3 and above	9.0 (6.3)		
Enacted stigma in community and civic life	Stage 2 and below	6.0 (5.7)	19.562	0.002
	Stage 3 and above	9.6 (9.6)		

Notes: Independent samples non-parametric analysis was performed using the Independent samples T-test; *SD, Standard deviation. *Stages I and II are rapidly reversible through adequate treatment, while stages III and above require long term treatment for improvement (Tekola et al. 2008).

## Discussion

Previous qualitative and quantitative studies have assessed the manifestations of podoconiosis-related stigma but have not quantified it using standardized instruments (GebreHanna 2005; Yakob et al. 2008; Tekola et al. 2009a; Tora et al. 2011; Molla et al. 2012). The present study measured and revealed high levels of stigma in Wolaita Zone. Over half of the affected individuals in endemic communities report significant levels of stigmatization. Interestingly, we did not find significant variation in the level of felt or enacted stigma by gender, age or education of the affected individuals. However, disease stage and level of stigma were strongly and positively associated which is congruent with a similar study in Northern Ethiopia (Deribe et al. 2013). This implies that being affected by the disease regardless of difference in socio-demographic attributes exposes a person to severe forms of stigmatization. As the disease progresses, it makes affected individuals economically less productive as it reduces their capacity to move and work (Desta et al. 2003; Tekola et al. 2006). More importantly, the social stigma related to podoconiosis is likely to be aggravated due to the non-concealable nature of swelling.

Prejudice and discrimination against podoconiosis patients were found to be high in major areas of life such as marriage and employment. For the majority of persons affected by podoconiosis, forced divorce and dissolution of marriage were very common. Deeply rooted misconceptions about the disease as contagious or caused by evil spirits and unstoppably transmitted through inheritance have been suggested to account for the stigmatizing attitude of community members and health professionals (Yakob et al. 2010; Ayode et al. 2012).

Felt stigma arises from the experience of severe forms of prejudice and discrimination (Scambler 2006; Weiss 2008). In this study, though felt stigma scores were slightly lower than enacted stigma scores, it was found to be the major burden particularly in interpersonal interactions. Avoidance of participation in social and group affairs and marriage with unaffected person due to fear of divorce and changing of place of residence were manifestations of felt stigma frequently mentioned. Notably, over half of the study participants said they had attempted suicide. A number of studies on the psychosocial consequences of health related stigma have also reported suicide ideation and attempt among the stigmatized individuals (Rafferty 2005; Sengupta et al. 2011; Singh 2012; Amiya et al. 2014). However, the reported level of suicidal attempt by the participants in this study within a 12-month timeframe is particularly concerning - an earlier study identified suicidal attempt among podoconiosis patients as one form of coping mechanism against stigma induced stress (Tora et al. 2011), but the extent of these attempts has not before been quantified. In the previous study, it has been stated that when prejudice and discrimination is directed against affected individuals by those who are close to them, they are exposed to a feeling of meaninglessness and hopelessness which forces them to consider suicide as a way out of the situation (Tora et al. 2011). Studies have suggested that social and family support are generally protective against adverse psychological responses to stressful situations (Cohen and Wills 1985), can have a direct beneficial impact on their health and wellbeing (Bekele et al. 2013) and buffer against suicide risk conferred by chronic disease and other stressful life events (Soykan et al. 2003). A recent study in Nepal also indicated that higher levels of perceived family support are strongly associated with reduced depression and suicidal intent (Amiya et al. 2014).

The present study also revealed that over 44% of the affected individuals felt their families were ashamed of their situation while 30% of them avoided sharing household utensils with other family members. This is congruent with earlier work in which some podoconiosis patients stated that they do not let their children use the same water or container they have used for washing their feet or let others in the family to wear their shoes thinking that they may develop the disease due to contact. Families of affected children also stop their children from going to clinics due to fear of public identification of a familial disease (Tora et al. 2012).

According to Scambler et al. (2006), felt stigma can prove to be a more disruptive factor than enacted stigma. As a direct consequence of discrimination, felt stigma creates a serious psychosocial burden on the stigmatized impacting low self-esteem (“I am not worthy”) and self-efficacy (“I am not able”) leading to self-blame, hopelessness and helplessness (Link and Phelan 2001; Heijnders and Van Der Meij 2006; Weiss 2008). It also pushes the stigmatized to adopt non-disclosure and concealment of the condition. This may make patients more susceptible to disability because their access to social, health and financial resources is reduced or curtailed (Scambler 2006; Weiss 2008; Tora et al. 2012). Felt stigma related to podoconiosis also has similar negative consequences on the psychosocial wellbeing of the affected individuals.

## Conclusions

Even though the small sample size and route of patient identification may have threatened the accuracy of estimation of the actual level of stigma, the implications of this study are that interventions against stigma are greatly needed. Though interventions in the form of disease prevention and control have been made for over a decade through the Mossy Foot International (formerly the Mossy Foot Treatment and Prevention Association) (see Davey and Burridge 2009), the nature and implications of these interventions towards reducing stigma have not yet been assessed. Similarly, treatment and control activities have also been started by faith-based organizations in western Ethiopia and northern Ethiopia. However, there is no evidence yet to support the effectiveness of those interventions in terms of stigma reduction. The nature of and challenges related to podoconiosis stigma reduction interventions have not yet been explored by these faith-based organisations.

Health-related stigma researchers have suggested multi-strategy and multi-level approaches to stigma reduction. These use intrapersonal, interpersonal and organizational strategies at community and governmental levels (Brown et al. 2003; Heijnders and Van Der Meij 2006; Cook et al. 2014). Since the recognition of podoconiosis as one of the Neglected Tropical Diseases by the WHO in 2011 and its prioritization by the Ethiopian Federal Ministry of Health in 2013, there is a window of opportunity for podoconiosis prevention and control programs in Ethiopia through which stigma reduction strategies could be implemented. The recent establishment of a consortium named the National Action Network for Podoconiosis (NaPAN) whose aim is to integrate interventions and standardize podoconiosis services is also an important step forward in podoconiosis prevention in Ethiopia. All these factors suggest it is the right time to introduce multi-strategy intervention programs in order to reduce podoconiosis stigma. Development of these programs should benefit from the experience of stigma reduction strategies proved to be effective for other diseases such as HIV/AIDS, leprosy and obesity.

## Methods

### Ethics statement

Following guidance from Tekola’s rapid ethical assessment done in the same area (Tekola et al. 2009b), podoconiosis patients were first asked to participate by staff of Mossy Foot International (MFI), a non-government organisation providing care and treatment to patients. Patients are familiar with MFI staff, who introduced the study team. The purpose of the study was explained orally, and potential participants gave their consent verbally, countersigned by a witness as confirmation. MFI staff members, many of whom are themselves treated patients, understand the psycho-social burdens of podoconiosis, in particular stigma, and are able to provide emotional support to those who may feel distressed answering the questionnaire. Care was taken to ensure participants knew their responses would be kept anonymous and confidential. Approval of this study, and specifically for this method of obtaining consent, was obtained from the Ethics Committee in Wolaita Sodo University and from the Research Governance and Ethics Committee at Brighton & Sussex Medical School.

### Study area

The study was conducted in Wolaita zone, southern Ethiopia, located 385 km from Addis Ababa, the capital of Ethiopia. Wolaita is one of the most densely populated areas in Ethiopia with an estimated total population of 1.7 million in 2007 (Central Statistical Agency (CSA) 2008). Most people earn their living from subsistence agriculture. The point prevalence of podoconiosis in this area has been calculated at 5.46% (Desta et al. 2003). In this podoconiosis-endemic area, Mossy Foot International (formerly, the Mossy Foot Treatment and Prevention Association), an international non-governmental association, offers community-based prevention and treatment at 15 outreach sites located 15 to 65 km from the head office in Sodo town (Davey and Burridge 2009).

### Study design and sampling

Assumptions used in the sample size estimate were drawn from Brieger and colleagues’ study measuring onchocerciasis (another NTD) stigma in Western Nigeria (Brieger et al. 1998), and the desired size of reduction in stigma level towards podoconiosis patients were an intervention to be introduced. Full details of sample size calculation are given in Franklin et al. 2013.

### Data collection

Data collection was conducted in May 2011, and included brief socio-demographic questions on age, gender and education, and assessment of disease stage. Based on the standardized clinical staging system of podoconiosis (Tekola et al. 2008), the clinic site health agents assessed the disease stage of patients recruited for the study.. We employed podoconiosis stigma assessment scales whose development and validation is described in another article (Franklin et al. 2013). The scales each measure the extent of felt and enacted stigma in three domains: interpersonal interactions, major life areas, and the community, social, and civic life of podoconiosis patients over the last 12 months.

The scales were translated to the local language *Wolaitigna* and administered through face-to-face interviews because of the low literacy of the respondents. Six data collectors with a BA degree or higher in social and behavioural sciences were hired and briefed on the purpose of the study, the eligibility criteria for participants and how to obtain consent and administer the questionnaires. The interviews were conducted in a private area of the Mossy Foot International outreach clinics where they were approached.

### Data analysis

Responses to the negatively framed items in the scale were rated from 3 to 0: 3 for a ‘yes’ response, 2 for a ‘possibly’ response, 1 for an ‘uncertain’ response and 0 for a ‘no’ response for each item. This coding was reversed for positively framed items. Each of the items received equal weight when summed, to arrive at a global (total) score: a low global score representing low stigma experience, a high score indicating high stigma experience. The mean scores (and standard deviations) were calculated for felt and enacted stigma and across the domains. To compare the distribution of mean scores of stigma by gender, level of education and disease stage categories, we used the Independent-Samples T Test. The associations between age categories and mean stigma scores were tested using the Kruskal-Wallis 1-way ANOVA (k samples) test. Significance level was determined at alpha level of 0.05 and below. Item-based descriptive analysis was conducted by transforming four response categories in the scale: ‘Yes’, ‘Possibly’, ‘Uncertain’ and ‘No’ into three taking ‘possibly’ as almost equivalent to ‘Yes’.
